# The prognostic value of the advanced lung cancer inflammation index in patients with gastrointestinal malignancy

**DOI:** 10.1186/s12885-023-10570-6

**Published:** 2023-01-30

**Authors:** Lilong Zhang, Kailiang Zhao, Tianrui kuang, Kunpeng Wang, Dongqi Chai, Zhendong Qiu, Rongqiang Liu, Wenhong Deng, Weixing Wang

**Affiliations:** 1 Department of General Surgery, Renmin Hospital of Wuhan University, Wuhan, China; 2 Central Laboratory, Renmin Hospital of Wuhan University, Wuhan, China; 3 Hubei Key Laboratory of Digestive System Disease, Wuhan, China

**Keywords:** Advanced lung cancer inflammation index, Gastrointestinal cancers, Meta-analysis, Prognosis

## Abstract

**Background:**

Systemic inflammation is crucial for the development and progression of cancers. The advanced lung cancer inflammation index (ALI) is considered to be a better indicator of systemic inflammation than current biomarkers. However, the prognostic value of the ALI in gastrointestinal neoplasms remains unclear. We performed the first meta-analysis to explore the association between ALI and gastrointestinal oncologic outcomes to help physicians better evaluate the prognosis of those patients.

**Methods:**

Eligible articles were retrieved using PubMed, the Cochrane Library, EMBASE, and Google Scholar by December 29, 2022. Clinical outcomes were overall survival (OS), disease-free survival (DFS), progression-free survival (PFS), and cancer-specific survival (CSS).

**Results:**

A total of 18 articles with 6898 patients were included in this meta-analysis. The pooled results demonstrated that a low ALI was correlated with poor OS (HR = 1.914, 95% CI: 1.514–2.419, *P* < 0.001), DFS (HR = 1.631, 95% CI: 1.197–2.224, *P* = 0.002), and PFS (HR = 1.679, 95% CI: 1.073–2.628, *P* = 0.023) of patients with gastrointestinal cancers. Subgroup analysis revealed that a low ALI was associated with shorter OS (HR = 2.279, 95% CI: 1.769–2.935,* P* < 0.001) and DFS (HR = 1.631, 95% CI: 1.197–2.224, *P* = 0.002), and PFS (HR = 1.911, 95% CI: 1.517–2.408, *P* = 0.002) of patients with colorectal cancer. However, the ALI was not related to CSS in the patients with gastrointestinal malignancy (HR = 1.121, 95% CI: 0.694–1.812, *P* = 0.640). Sensitivity analysis supported the stability and dependability of the above results.

**Conclusion:**

The pre-treatment ALI was a useful predictor of prognosis in patients with gastrointestinal cancers.

## Introduction

Gastrointestinal cancers (GIC) account for over one-quarter of all cancer cases and one-third of cancer-associated deaths worldwide [1]. Although there has been great advancement in the treatment of GIC, the outcome for the majority of GIC patients remains poor [2]. Thus, exploring a reliable prognostic index for patient survival can enable physicians to adopt better therapeutic and preventative measures.

Numerous studies in recent years have confirmed that systemic inflammation is crucial for the development and growth of GIC [3, 4]. A variety of inflammatory cells and proinflammatory cytokines are activated in the early stages of carcinogenesis, which promote the creation of lymphatic ducts and new blood vessels, causing a pro-cancer microenvironment for growth and differentiation [5]. At later stages, cancer-related inflammation can impair immune cell function, creating a conducive environment for metastasis [6]. Thus, inflammatory indicators are anticipated to be important prognostic biomarkers in cancer. For instance, an elevated neutrophil-to-lymphocyte ratio (NLR) is linked to a weak immunological response and a high inflammatory response [7–9]. In cancer patients, the nutritional status of the body is also closely associated with tumor development and clinical outcome. Some common nutritional indicators have been shown to have a high prognostic significance in cancer, such as body mass index (BMI) [10] and serum albumin level [11].

Recently, the advanced lung cancer inflammation index (ALI), a new inflammatory marker that is calculated as BMI (kg/m2) × albumin (g/dL)/NLR, was initially found to be a useful prognostic index in lung cancer [12]. ALI is thought to reflect systemic inflammation better than other biomarkers due to combining the indicators of nutrition and inflammation. To date, some retrospective articles have analyzed the association between ALI and prognosis in GIC patients. However, there has not been a systematic evaluation of whether ALI is a reliable predictive factor for GIC patients. Thus, we conducted the first meta-analysis to identify the predictive significance of pre-treatment ALI in GIC patients, which may help to determine prognosis and formulate an effective treatment strategy that will further minimize mortality.

## Methods

### Literature search strategies

The current meta-analysis accompanied the PRISMA statement [13]. The protocol for this meta-analysis was available in PROSPERO (CRD42022371374). On December 29, 2022, PubMed, EMBASE, and the Cochrane Library were retrieved using the keyword: “advanced lung cancer inflammation index [All Fields]”. We further searched Google Scholar for grey literature. Additionally, we manually retrieved the reference lists of the publications that qualified.

### Inclusion and exclusion criteria

If studies met all the following criteria, they were included: patients diagnosed with GIC; research evaluated the prognostic value of ALI; provided at least one of the outcomes [overall survival (OS), disease-free survival (DFS), progression-free survival (PFS), and cancer-specific survival (CSS)]. The conference abstracts, case reports, or comments were excluded.

### Data extraction and quality assessment

Data extraction mainly focused on the author, year, study region, study design, study period, sample size, the number of male and female patients, cancer types, treatment, follow-up duration, cut-off, and outcomes. The Newcastle–Ottawa Scale (NOS) score was utilized to evaluate the quality of the observational studies. High-quality literature was defined as having a score above six. All of the above steps were double-checked by Lilong Zhang and Kailiang Zhao, and any disparities were addressed by Weixing Wang and Wenhong Deng.

### Statistical methods

Statistical analysis was conducted by Stata 15.0. The statistical heterogeneity was calculated using the chi-squared test. *P* < 0.1 and I^2^ > 50% indicated high heterogeneity, so a random effect model was applied; otherwise, the fixed effect model was used. The tests of Egger’s and Begg’s were employed to evaluate publication bias. If there was significant publication bias, we used the trim-and-fill method to modify the results [14]. Sensitivity analysis was implemented to assess the stability of the results by excluding each study independently.

## Results

### Characteristics of studies

**Fig. 1:**
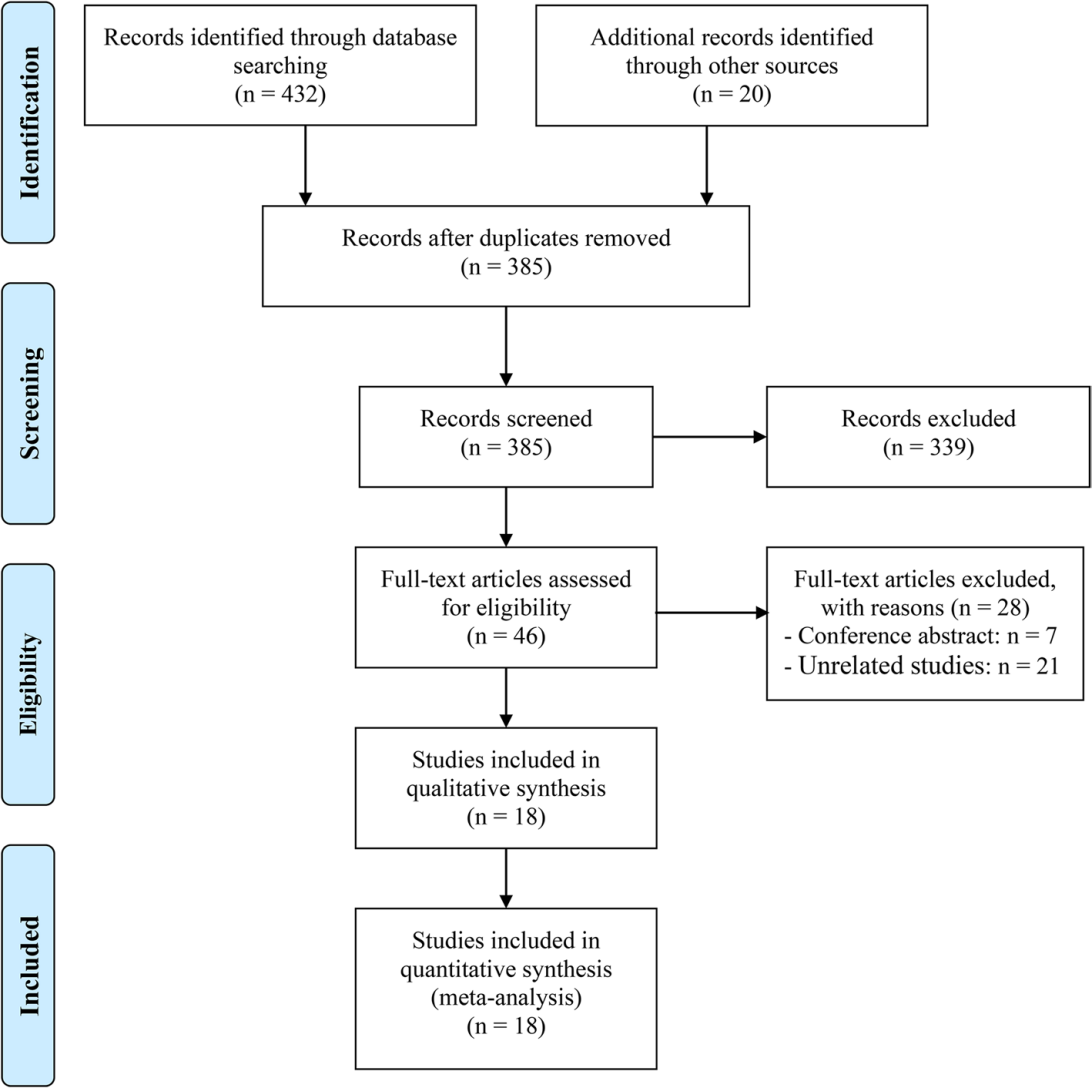
The flow diagram of identifying eligible studies

After the initial search, 67 duplicate studies were removed. Then there were 339 articles deleted after carefully reading the titles and abstracts. Later, the full texts of the remaining 46 articles were further assessed. 18 articles involving 6899 patients were ultimately included [15–32]. The PRISMA flow diagram is provided in Fig. 1.

The main characteristics of the studies included are shown in Table 1. Of the 18 studies, seven were on colorectal cancer (CRC), three on gastric cancer (GC), two on esophageal cancer (EC), two on pancreatic cancer (PC), one on hepatocellular carcinoma (HCC), one on oral cavity cancer (OCC), and one on cholangiocarcinoma (CC). Besides, the study by Ruan et al*.* included 270 patients with CRC, 245 patients with GC, 145 patients with EC, and 31 patients with hepatobiliary cancer. Eleven studies were carried out in China and four in Japan, plus one each in Austria, Turkey, and Korea. The cutoff point of the ALI was reported as ranging from 13.2–70.4. The NOS scores for 18 articles ranged from 6–8, which represented a low risk of bias (Table 1).

**Table 1:** Main characteristics of the studies included

Study	Study region	Study design	Study period	Sample size	Male/ Female	Cancer types	Treatment	Follow-up duration	Cut-off	Out-come	NOS
Zhang et al. 2022 [15]	Xi'an, China	R	2010- 2017	615	469/146	GC	Surgery	-	39.8	OS (M) DFS (M)	8
Horino et al. 2022 [18]	Kumamoto, Japan	R	04/2005–06/2019	813	464/349	CRC	Surgery	-	43.1/13.2^e^	OS (M) DFS (M)	7
He et al. 2022 [19]	Nanjing, China	R	01/2009–03/2016	358	284/74	GC	Surgery	101 (2–166)^a^	40.5	OS (M)	6
Deng et al. 2022 [20]	Fuzhou, China	R	01/2012–12/2016	360	194/166	CRC	Surgery	65 (3–110)^a^	36.3	OS (M) DFS (M)	7
Tan et al. 2022 [23]	Nanning, China	R	09/2013–07/2018	158	126/32	EC	Surgery	-	31.2	OS (M)	6
Xie et al. 2022 [25]	Nanning, China	R	2012- 2014	662	408/254	CRC	Surgery	63 (1–80)^a^	31.6/24.4^e^	OS (M) PFS (U)	7
Kusunoki et al. 2022 [26]	Mie, Japan	R	02/2005–11/2011	298	171/127	CRC	Surgery	39.7 ± 29.0^c^	20.5	OS (M) DFS (M)	6
Pian et al. 2022 [17]	Suwon, Korea	R	06/2009–06/2018	132	88/44	CRC	Surgery	-	70.4	OS (M) DFS (U)	7
Ruan et al. 2022 [24]	Multicenter, China	P	06/2012–12/2019	691	-	GM	Cancer Sarcopenia	43.7^b^	18.4	OS (M)	8
Wu et al. 2022 [16]	Fuzhou, China	R	2016- 2019	97	58/39	CC	Surgery	20 (3–70)^a^	31.8	OS (M) DFS (M)	6
Qian et al. 2022 [31]	Zhengzhou, China	R	2017- 2020	65	55/10	HCC	Camrel-izumab	-	34.7	OS (M)	6
Chen et al. 2022 [32]	Fuzhou, China	R	01/2013–04/2019	636	385/281	CRC	Surgery	59.3(40.6–80.4)^d^	40.0	OS (M)	7
Tsai, et al. 2021 [22]	Taiwan, China	R	01/2008–12/2017	372	336/36	OCC	Surgery	58.5 (2–126)^a^	33.6	OS (M) DFS (M)	6
Yin et al. 2021 [21]	Mie, Japan	R	1992–2011	620	424/196	GC	Surgery	52.8 ± 39.9^c^	30.0	OS (M)	8
Shibutani et al. 2019 [29]	Osaka, Japan	R	2008–2016	159	87/72	CRC	Chemo-therapy	21.6 (1.2–94.0)^a^	28.9	OS (M)	7
Topkan et al. 2019 [28]	Adana, Turkey	R	01/2007–12/2017	141	111/30	PC	Chemor-adiotherapy	14.4 (3.2–74.2)^a^	25.3	OS (U) PFS (U)	7
Barth et al. 2019 [27]	Graz, Austria	R	12/2003–10/2015	429	236/193	PC	Surgery or chemotherapy	-	43.5	CSS (M)	7
Feng et al. 2014 [30]	Hangzhou, China	R	01/2006–12/2008	293	259/34	EC	Surgery	-	18.0	CSS (M)	6

*P* Prospective cohort study, *R* Retrospective study, *GC* Gastric cancer, *CRC* Colorectal cancer, *EC* Esophageal cancer, *HCC* Hepatocellular carcinoma, *PC* Pancreatic cancer, *CC* Cholangiocarcinoma, *OCC* Oral cavity cancer, *GM* Gastrointestinal malignancy, including 245 patients with gastric cancer, 270 patients with colorectal cancer, 145 patients with esophageal cancer, and 31 patients with hepatobiliary cancer. *M* Multivariate analysis, *U* Univariate analysis

^a^medians with ranges

^b^medians

^c^Mean ± standard deviation

^d^median and interquartile range

^e^male/female

### ALI and overall survival

**Fig. 2:**
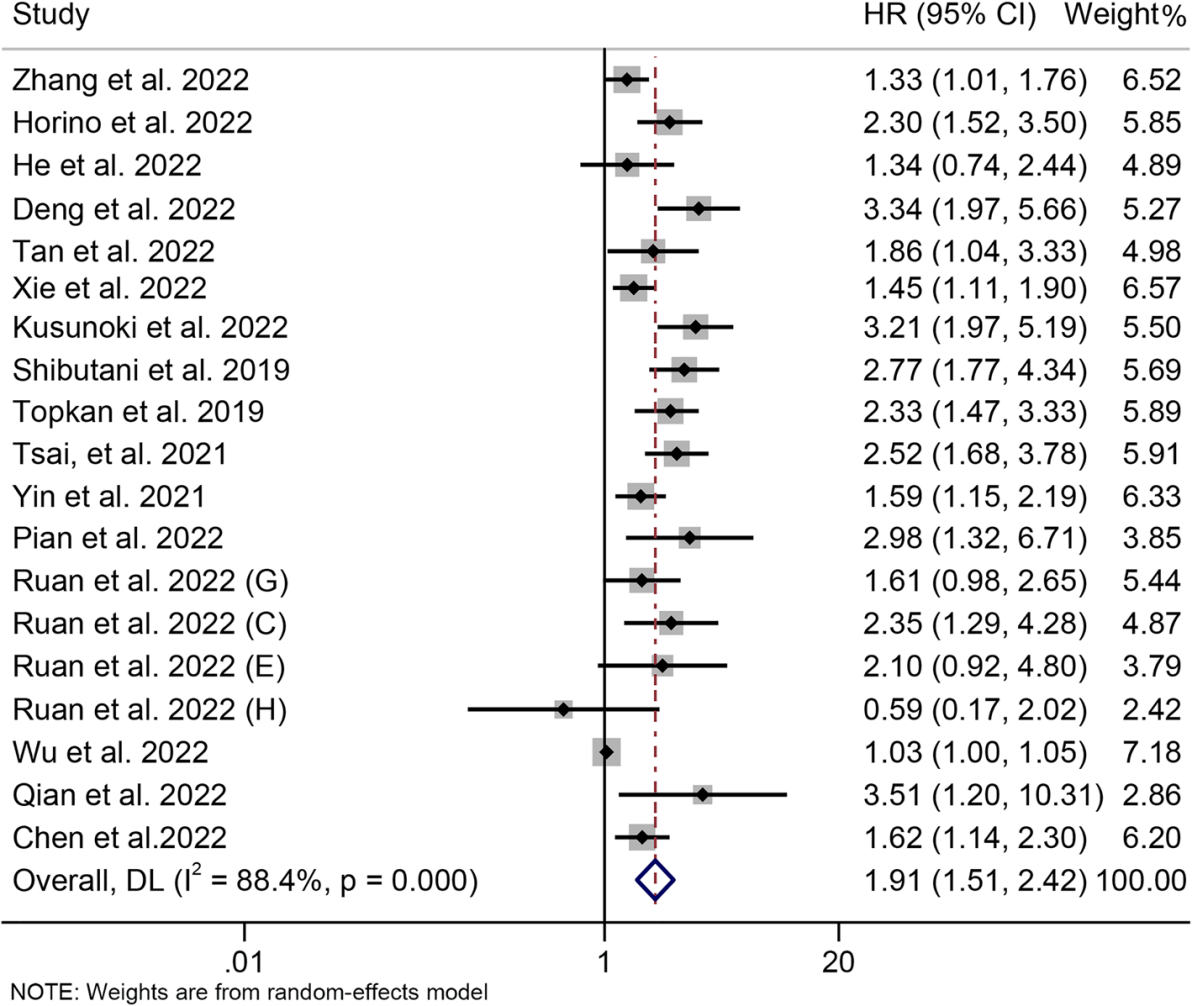
Forest plot of the advanced lung cancer inflammation index in relation to overall survival. HR, hazard ratio; CL, confidence interval

In total, 16 articles involving 6177 patients explored the association between ALI and OS in cancer patients. The pooled HR was 1.914 (95% CI: 1.514–2.419, *P* < 0.001), implying that low ALI raised death risk by 91.4% (Fig. 2). Since there was significant heterogeneity, a random effects model was used (I^2^ = 88.4%, *P* < 0.001).

**Fig. 3:**
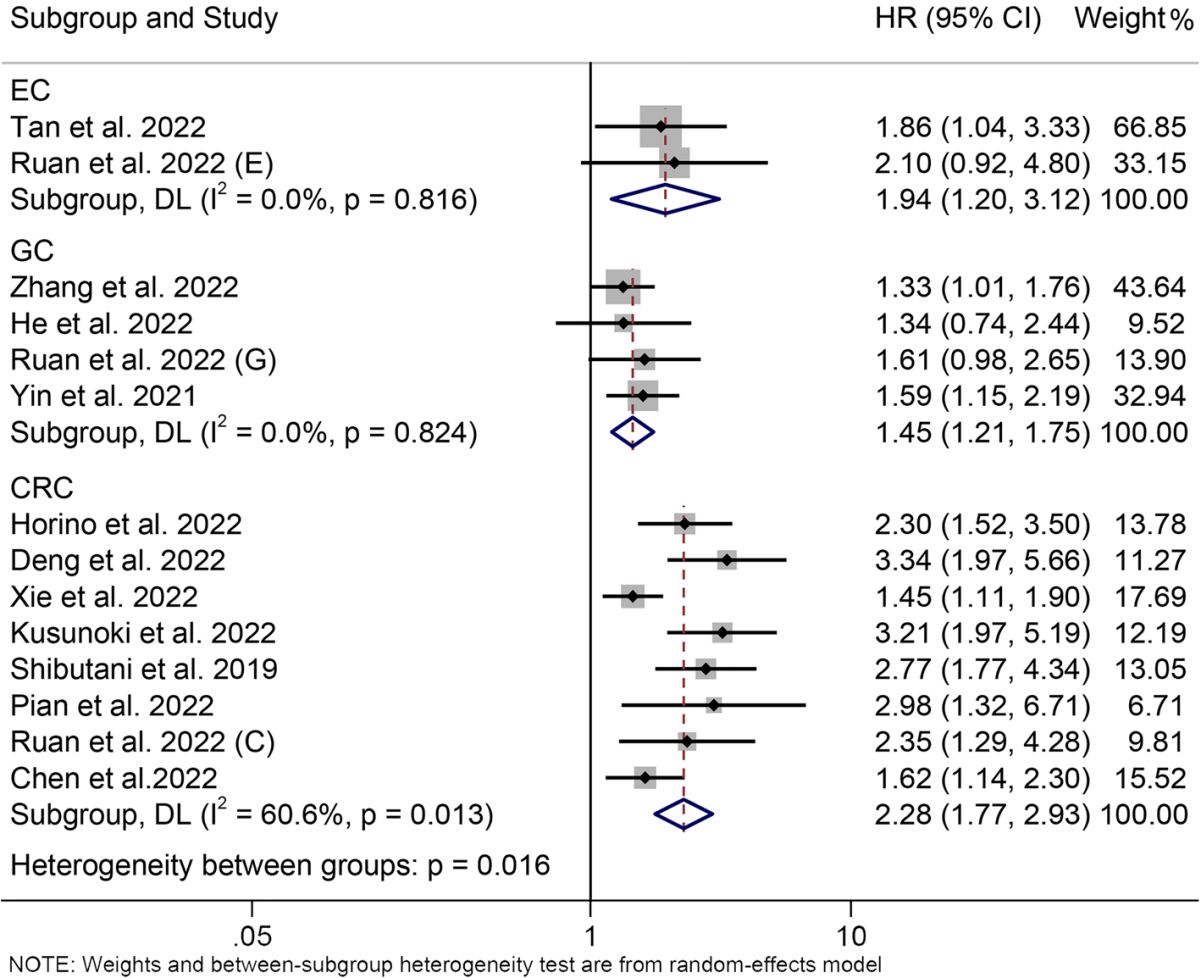
Subgroup analysis of overall survival based on cancer types. HR, hazard ratio; CL, confidence interval; EC, esophageal cancer; GC, gastric cancer; CRC, colorectal cancer

We then conducted subgroup analyses based on cancer types. The results showed that patients with low ALI had worse OS than those with high ALI in EC (HR = 1.937, 95% CI: 1.204–3.119, *P* = 0.006, Fig. 3), GC (HR = 1.451, 95% CI: 1.206–1.746, *P* < 0.001, Fig. 3), and CRC (HR = 2.279, 95% CI: 1.769–2.935,* P* < 0.001, Fig. 3). We also found no significant heterogeneity between included studies in the GC (I^2^ = 0.0%, *P* = 0.824) and EC (I^2^ = 0.0%, *P* = 0.816) subgroups; and lower heterogeneity between included studies in the CRC (I^2^ = 60.6%, *P* = 0.013) subgroup, so differences in cancer type were a source of heterogeneity (Fig. 3).

Subgroup analyses based on study region, sample size, treatment, and ALI cutoff were also performed, and the results for each subgroup were consistent with the above findings (Table 2).

**Table 2:** Subgroup analysis of the association between ALI and overall and disease-free survival in patients with gastrointestinal neoplasms

Variable	Included studies	Test of association			Test of heterogeneity		
		HR	95%CI	*P* value	Modal	I^2^	*P* value
Overall survival							
Study region							
China	10	1.694	1.316–2.180	< 0.001	RE	84.3%	< 0.001
Japan	4	2.313	1.676–3.192	< 0.001	RE	59.0%	0.063
Other	2	2.444	1.696–3.523	< 0.001	RE	0.0%	0.596
Sample size							
≤ 300	8	2.016	1.364–2.980	< 0.001	RE	88.1%	< 0.001
> 300	8	1.777	1.448–2.182	< 0.001	RE	58.9%	0.017
Treatment							
Surgery	12	1.837	1.406–2.402	< 0.001	RE	89.5%	< 0.001
Other	2	2.156	1.658–2.804	< 0.001	RE	23.0%	0.253
Cut-off							
> 30	9	1.839	1.320–2.564	< 0.001	RE	87.3%	< 0.001
≤ 30	5	2.086	1.627–2.674	< 0.001	RE	45.5%	0.076
Other	2	1.776	1.136–2.775	0.012	RE	69.7%	0.069
Disease-free survival							
Study region							
China	4	1.562	1.047–2.332	0.029	RE	89.6%	0.526
Japan	2	1.838	1.373–2.461	< 0.001	RE	0.0%	< 0.001
Korea	1	1.456	0.812–2.604	0.206	-	-	-
Sample size							
≤ 300	3	1.385	0.859–2.233	0.182	RE	76.4%	0.014
> 300	4	1.783	1.369–2.322	0.000	RE	52.2%	0.099
Cut-off							
> 30	1	2.130	1.240–3.659	0.006	-	-	-
≤ 30	5	1.540	1.081–2.193	0.017	RE	86.8%	< 0.001
Other	1	1.730	1.223–2.447	0.002	-	-	-

### ALI and disease-free survival

**Fig. 4:**
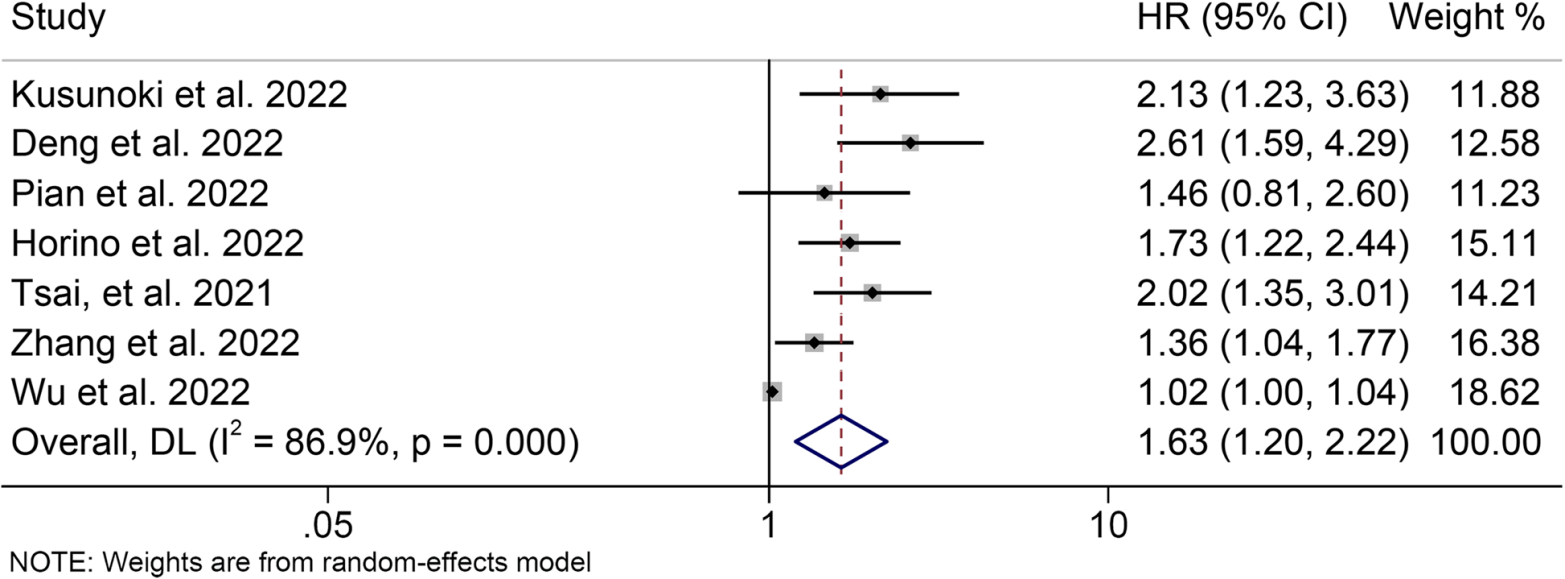
Forest plot of the advanced lung cancer inflammation index in relation to disease-free survival. HR, hazard ratio; CL, confidence interval

The relationship between ALI and DFS was also examined using prognostic data from 7 studies involving 3,047 participants. Significant heterogeneity was observed in the included studies (I^2^ = 86.9%, *P* < 0.001, Fig. 4), so a random effects model was used. We found that patients with low ALI had a shorter DFS than those with high ALI (HR = 1.631, 95% CI: 1.197–2.224, *P* = 0.002, Fig. 4).

**Fig. 5:**
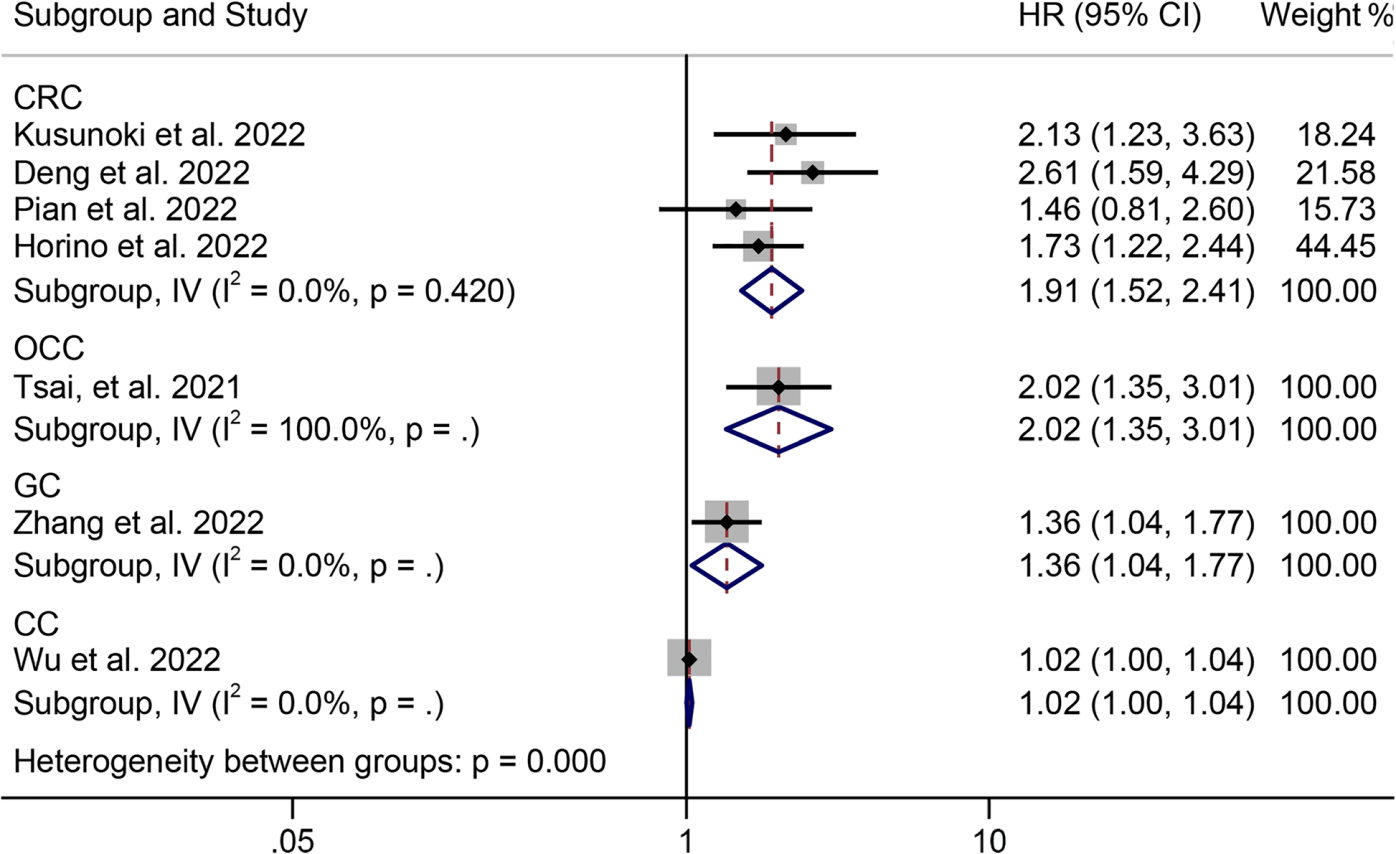
Subgroup analysis of disease-free survival based on cancer types. HR, hazard ratio; CL, confidence interval; CC, cholangiocarcinoma; GC, gastric cancer; CRC, colorectal cancer; OCC, oral cavity cancer

Subgroup analysis showed that lower ALI was associated with poorer DFS in CRC patients (HR = 1.911, 95% CI: 1.517–2.408, *P* = 0.002, Fig. 5). No significant heterogeneity was observed in the subgroups (I^2^ = 0.0%, *P* = 0.420, Fig. 5), and a fixed effects model was utilized. Therefore, differences in cancer type were the source of heterogeneity.

In addition, subgroup analyses based on study region, sample size, and ALI cutoff were also performed, and the results for each subgroup were generally consistent with the above results (Table 2). Notably, ALI was not found to be associated with worse DFS in subgroups with sample sizes ≤ 300 (HR = 1.385, 95% CI: 0.859–2.233,* P* = 0.182); the opposite was true in subgroups with sample sizes > 300 (HR = 1.783, 95% CI: 1.369–2.322, *P* < 0.001).

### ALI and progression-free survival and cancer-specific survival

**Fig. 6:**
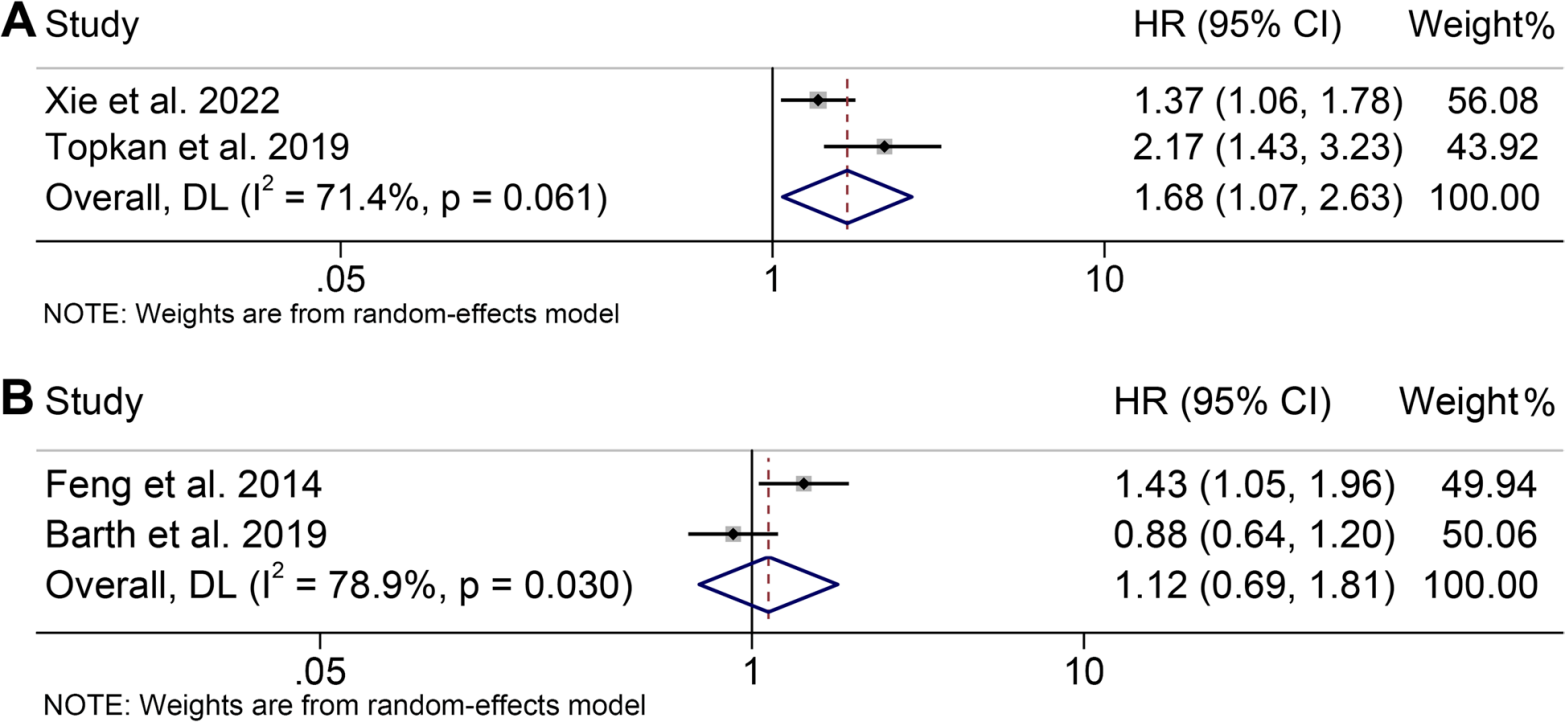
Forest plot of the advanced lung cancer inflammation index in relation to progression-free survival (**A**) and cancer-specific survival (**B**). HR, hazard ratio; CL, confidence interval

A connection between ALI and PFS in cancer patients was observed in a total of 2 studies involving 803 individuals. As shown in Fig. 6A, patients with low ALI had a worse PFS than those with high ALI (HR = 1.679, 95% CI: 1.073–2.628, *P* = 0.023). Significant heterogeneity was found in studies (I^2^ = 71.4%, *P* = 0.061), and a random effects model was applied to this analysis.

The association between ALI and CSS in cancer patients was explored in two articles with 722 individuals (Fig. 6B). Interestingly, we found no significant correlation between ALI and CSS in cancer patients (HR = 1.121, 95% CI: 0.694–1.812, *P* = 0.640) using a random effects model (I^2^ = 78.9%, *P* = 0.030).

### Sensitivity analysis

**Fig. 7:**
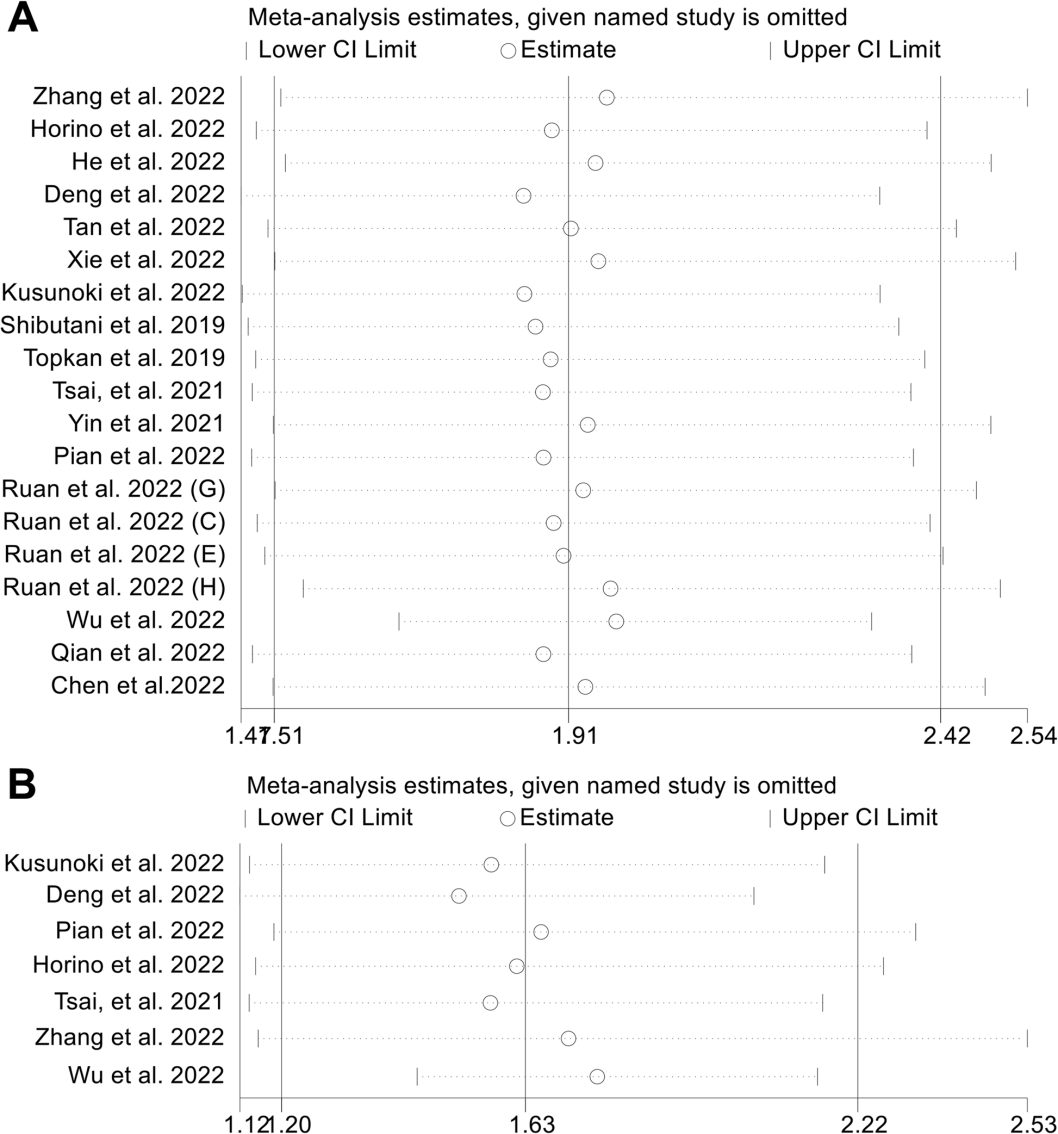
Sensitivity analysis of overall survival (**A**) and disease-free survival (**B**). CL, confidence interval

We used the leave-one-out method to do a sensitivity analysis to assess how each study might impact the combined results. We found that the pooled HR for OS was not significantly changed after excluding one study at a time, ranging from 1.853 (95% CI: 1.469–2.337, after omitting Deng et al*.* 2022) to 1.966 (95% CI: 1.523–2.537, after omitting Zhang et al*.* 2022, Fig. 7A). Similarly, the pooled HR for DFS was not significantly different in the sensitivity analysis (Fig. 7B). The overall HR ranged from 1.513 (95% CI: 1.123–2.039, after omitting Deng et al*.* 2022) to 1.708 (95% CI: 1.155–2.526, after omitting Zhang et al*.* 2022). From the above, we can see that our results are stable and reliable.

### Publication bias

The publication bias in OS (Egger’s test: *P* = 0.001, Begg’s test: *P* = 0.548) and DFS (Egger’s test: *P* = 0.021, Begg’s test: *P* = 0.548) was found by Egger's test. Next, the trim and fill method was utilized to calculate the number of missing studies in OS and DFS. By factoring in the missing hypothesis studies, the combined HR of OS and DFS was recalculated but was not substantially different. As a result, the publication bias had little impact, and the outcome was stable.

## Discussion

Our goal was to explore the predictive significance of ALI in GIC patients, and the pooled data demonstrated that a lower ALI was remarkably related to shorter OS, DFS, and PFS. Furthermore, these results held steady even after sensitivity analysis and subgroup analysis. This is the first meta-analysis to thoroughly explore the impact of ALI on the prognosis of GIC patients. As an extremely accessible indicator in clinical practice, pre-treatment assessment of patients’ ALI can help physicians more effectively and easily predict clinical outcomes and assist them to adjust treatment in a timely manner, thereby further reducing mortality. However, it is worth noting that our results also found that ALI levels were not associated with CSS in patients with GIC. Considering that this index (including PFS) only integrated the data of two studies, it may lead to instability in the results, which need to be further confirmed by subsequent studies.

Both the systemic inflammatory response and nutritional state are recognized prognostic factors in cancer patients, and mounting research has shown a close relationship between the systemic inflammatory response and nutritional status in various cancers [33]. Furthermore, the latest view is that systemic inflammatory response via host-tumor interaction is now considered to be the 7th hallmark of cancer [34]. Systemic inflammatory response and nutritional status have been assessed using a variety of blood examination-based derivatives up to this point, such as NLR [35], platelet-lymphocyte ratio (PLR) [36, 37], prognostic nutrition index (PNI) [38], BMI [39], and albumin [40], and a number of lines of research have shown that these derivatives have the potential to be employed by patients with malignancies as prognostic markers [35–40].

The ALI is a newly defined cancer index, and one of its unique features is as a composite index combining the nutritional state and the inflammatory state [12]. Deng et al*.* confirmed the predictive ability of the ALI for 5-year OS and 5-year DFS was better than that of the PNI or systemic inflammation index (SII) in CRC patients [20]. Some studies also found that ALI was superior to albumin, NLR, and BMI in predicting complications, 5-year PFS, and 5-year OS in CRC and OCC patients [17, 22]. Interestingly, Wu et al*.* revealed that ALI outperformed NLR, PLR, monocyte-lymphocyte ratio (MLR), SII, and PNI in predicting OS and DFS in patients with cholangiocarcinoma by using time-dependent ROC analysis [16]. Thus, the ALI may have a higher discriminating value compared to other biomarkers. Taking all the current evidence together, our study found that ALI predicted a poor prognosis in patients with GIC, and the results held true in gastric, oesophageal, and colorectal cancers, according to subgroup analysis.

Surely, this analysis still has some limitations. The absence of ALI dynamics' evaluations, rather than the use of a single time-point value, is a significant limitation. The absence of a correlation between interleukins, chemokines, and ALI prevents us from elucidating the mechanistic relationship between ALI values and clinical outcomes. The use of various salvage maneuvers may, by chance, have altered the results in favor of one group depending on the opportunities at the treatment center. The vast majority of articles were retrospective cohort studies, which possibly limited their statistical power. There is a lack of uniformity in the cut-off values for ALI across studies, and aggregated survival results may deviate from the actual values. Thus, in order to confirm and update our conclusion, more high-quality studies with sizable sample sizes, particularly multicentre RCTs, were urgently required. At the same time, these studies should also include patients of different races and explore the optimal cut-off values to guide the clinic more precisely for the benefit of patients.

## Data Availability

The data that support the findings of this study are available from the corresponding author upon reasonable request.
